# Establishment of an in vitro model for analyzing mitochondrial ultrastructure in *PRKN*-mutated patient iPSC-derived dopaminergic neurons

**DOI:** 10.1186/s13041-021-00771-0

**Published:** 2021-03-23

**Authors:** Mutsumi Yokota, Soichiro Kakuta, Takahiro Shiga, Kei-ichi Ishikawa, Hideyuki Okano, Nobutaka Hattori, Wado Akamatsu, Masato Koike

**Affiliations:** 1 Department of Cell Biology and Neuroscience, Juntendo University Graduate School of Medicine, 2-1-1 Hongo, Bunkyo-ku, Tokyo, 113-8421 Japan; 2 Laboratory of Morphology and Image Analysis, Research Support Center, Juntendo University Graduate School of Medicine, Tokyo, 113-8421 Japan; 3 Department of Cellular and Molecular Neuropathology, Juntendo University Graduate School of Medicine, Tokyo, 113-8421 Japan; 4 Center for Genomic and Regenerative Medicine, Juntendo University Graduate School of Medicine, Tokyo, 113-8421 Japan; 5 Department of Neurology, Juntendo University School of Medicine, Tokyo, 113-8421 Japan; 6 Department of Physiology, Keio University School of Medicine, Tokyo, 160-8582 Japan; 7 Advanced Research Institute for Health Sciences, Juntendo University, Bunkyo, Tokyo, 113-8421 Japan

**Keywords:** Mitochondria, Ultrastructure, PRKN, IPSC, Dopaminergic neurons

## Abstract

Mitochondrial structural changes are associated with the regulation of mitochondrial function, apoptosis, and neurodegenerative diseases. *PRKN* is known to be involved with various mechanisms of mitochondrial quality control including mitochondrial structural changes. Parkinson’s disease (PD) with *PRKN* mutations is characterized by the preferential degeneration of dopaminergic neurons in the substantia nigra pars compacta, which has been suggested to result from the accumulation of damaged mitochondria. However, ultrastructural changes of mitochondria specifically in dopaminergic neurons derived from iPSC have rarely been analyzed. The main reason for this would be that the dopaminergic neurons cannot be distinguished directly among a mixture of iPSC-derived differentiated cells under electron microscopy. To selectively label dopaminergic neurons and analyze mitochondrial morphology at the ultrastructural level, we generated control and *PRKN*-mutated patient tyrosine hydroxylase reporter (TH-GFP) induced pluripotent stem cell (iPSC) lines. Correlative light-electron microscopy analysis and live cell imaging of GFP-expressing dopaminergic neurons indicated that iPSC-derived dopaminergic neurons had smaller and less functional mitochondria than those in non-dopaminergic neurons. Furthermore, the formation of spheroid-shaped mitochondria, which was induced in control dopaminergic neurons by a mitochondrial uncoupler, was inhibited in the *PRKN*-mutated dopaminergic neurons. These results indicate that our established TH-GFP iPSC lines are useful for characterizing mitochondrial morphology, such as spheroid-shaped mitochondria, in dopaminergic neurons among a mixture of various cell types. Our in vitro model would provide insights into the vulnerability of dopaminergic neurons and the processes leading to the preferential loss of dopaminergic neurons in patients with *PRKN* mutations.

**Supplementary Information:**

The online version contains supplementary material available at 10.1186/s13041-021-00771-0.

## Introduction

*PRKN/PARK2*, which encodes Parkin RBR E3 ubiquitin protein ligase, is one of the most common genes responsible for early-onset familial Parkinson’s disease (PD). Parkin regulates mitochondrial quality control, such as mitochondrial fission/fusion, transport, biogenesis, and mitophagy [1–3]. The accumulation of damaged mitochondria caused by Parkin dysfunction has been suggested to contribute to pathogenesis in PD [4, 5]. To elucidate the detailed mechanisms of PD development, Parkin knockout mouse models have been generated in previous studies [6–10]. Nevertheless, these Parkin knockout mice have reproduced only some parts of the PD phenotypes [6–9, 11]. In contrast, in patient-specific induced pluripotent stem cell (iPSC)-derived neurons, *PRKN* mutations have been reported to result in abnormal mitochondrial morphology and a failure of mitochondrial degradation [12–14], an inability to form complex neuronal morphology [15], and reduced dopamine use [16].

It has recently been reported that Parkin is associated with various types of mitochondrial structural changes, for example spheroid-shaped mitochondria [17, 18], mitochondrial-derived vesicles [19, 20], and the endoplasmic reticulum (ER)-mitochondria interface [21]. These structural changes in mitochondria have been considered as mechanisms of mitochondrial quality control under mitochondrial stress. To detect such structures, ultrastructural analyses of mitochondria are essential. However, it remains unknown whether the mitochondrial structural changes are caused by mitochondrial stress in PD patients with *PRKN* mutations, because there is a scarcity of ultrastructural studies in iPSC-derived neurons from PD patients.

Furthermore, considering that PD with *PRKN* mutations is characterized by the preferential loss of dopaminergic neurons in the substantia nigra pars compacta, ultrastructural changes of mitochondria should be analyzed specifically in dopaminergic neurons derived from iPSC. However, dopaminergic neuron-specific ultrastructural analysis by conventional electron microscopy proven difficult. The main reason for this would be that the dopaminergic neurons cannot be distinguished directly among a mixture of iPSC-derived differentiated cells under electron microscopy. Although the efficiency of differentiation from iPSCs into dopaminergic neurons has been improved in recent studies, it remains at approximately 30–40% [13, 14, 22]. It is therefore necessary to selectively label dopaminergic neurons among the mixture of dopaminergic and non-dopaminergic neurons that are derived from iPSCs. Recently, several studies have reported the successful purification of dopaminergic neurons carrying a tyrosine hydroxylase (TH; a marker for identifying dopaminergic neurons) knock-in reporter, either using the genome editing technology TALEN (transcription activator-like effector nuclease) or CRISPR/Cas9 systems [23–25]. However, the generation of TH reporter iPSC lines derived from PD patients, including those with *PRKN* mutations, has not been reported.

To label iPSC-derived dopaminergic neurons and analyze mitochondrial morphology in labelled dopaminergic neurons at the ultrastructural level, we generated control and *PRKN*-mutated patient TH reporter iPSC lines using the CRISPR/Cas9 system. With the generated reporter iPSC lines, we then analyzed mitochondrial morphology in reporter-positive dopaminergic neurons under normal conditions and CCCP treatment, using correlative light-electron microscopy (CLEM). CLEM is a powerful tool that provides both the locations of target proteins and/or cells and fine structural information by taking fluorescent images and the subsequent ultrastructural analysis [26, 27].

In the current study, by combining live cell imaging and CLEM of labeled dopaminergic neurons, we revealed smaller and less functional mitochondria in dopaminergic neurons compared with non-dopaminergic neurons. Moreover, we discovered mitochondrial ultrastructural changes under CCCP treatment which occurred prior to cell death and were impaired only in *PRKN*-mutated dopaminergic neurons. These results indicate that our TH reporter iPSC lines may be useful for comparing mitochondrial ultrastructure between control and *PRKN*-mutated dopaminergic neurons. Our findings offer insights into the vulnerability of dopaminergic neurons and the mechanisms of PD development.

## Methods

### Single guide RNA (sgRNA) vector design

**Fig. 1:**
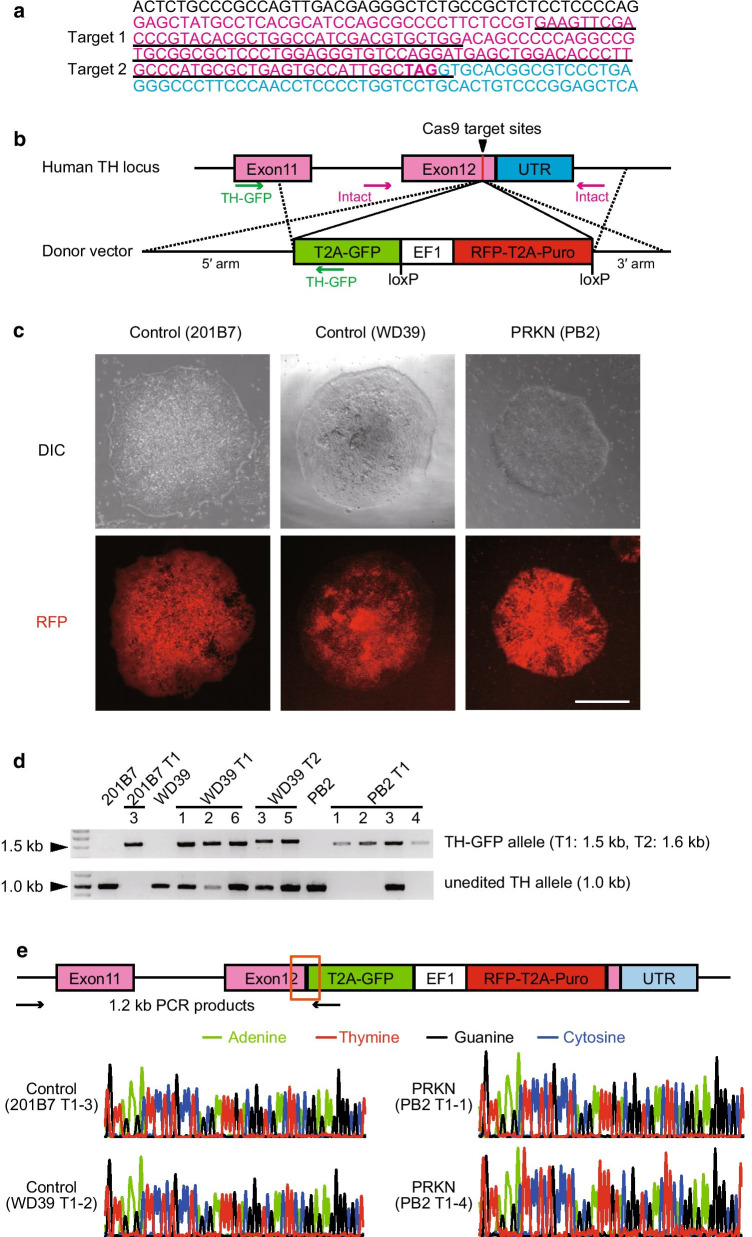
Generation of control and *PRKN*-mutated patient TH-GFP iPSC lines via the CRISPR/Cas9 system. **a** Cas9 target sites in the exon 12 sequence of the human *TH* gene. Black, magenta, and cyan characters represent intron, exon, and UTR sequences in the human *TH* gene, respectively. The stop codon is shown in bold. **b** Scheme describing the insertion of the reporter cassette into the *TH* gene by homologous recombination. Green and magenta arrows indicate the primer pairs used for the detection of the TH-GFP and unedited alleles, respectively. **c** The differential interference contrast (DIC) and fluorescent images showed RFP-positive knock-in iPS colonies after 8–9 days of puromycin selection. “PRKN” represents *PRKN*-mutated patient. Scale bar, 500 µm. **d** PCR analysis of knock-in iPS clones with TH-GFP alleles to determine whether the clones were homozygous or heterozygous for the TH-GFP allele. The TH-GFP allele produced by cleavage at the Target 1 (T1) or Target 2 (T2) site was found as a 1.5 kb or 1.6 kb band, respectively. The unedited TH allele was found as a 1.0 kb band. **e** Sanger sequencing confirmed that the donor sequence was correctly inserted into exon 12 of the *TH* gene in four knock-in iPSC lines. The 1.2 kb PCR products by primers (black arrows) were sequenced, and the sequence of the junction of the 5′ homology arm and T2A-GFP at the cleavage site (boxed area) is indicated as the electropherograms. “PRKN” represents *PRKN*-mutated patient

The Cas9 target sites were determined by entering the exon 12 sequence of tyrosine hydroxylase (NC_000011.10) into the CRISPR design tool (http://crispr.mit.edu/) [28]. A Target 1 site (Fig. 1a) was selected due to the highest score among candidates given by CRISPR design tool. A Target 2 site (Fig. 1a) was selected because it was close to the stop codon in the *TH* gene. The PCR products by forward primer including the Target 1 or 2 sites (Additional file 1: Table S1) and the Universal-reverse primer [29] were inserted into an RiH vector, which was a gift from Dr. Akitsu Hotta at Kyoto University (Addgene plasmid #60601) [29].

### Donor vector design for knock-in experiments

To insert a donor vector into genomic DNA by homologous recombination, 5′ or 3′ homology arms (0.9–1.1 kb) flanking the Cas9 target sites were amplified using primers (Additional file 1: Table S1) from genomic DNA of the 201B7 iPSC line [30]. The PCR fragments of 5′ or 3′ homology arms were inserted into EcoRI and BamHI cutting sites, respectively, of the HR130PA-1 vector (T2A-GFP-pA-loxP-EF1α-RFP-T2A-Puro-pA-LoxP-MCS, System Biosciences) using In-Fusion technology.

### Human iPSCs

Three previously reported iPSC lines were used for the knock-in experiments. 201B7, for a control line, was kindly provided by Dr. Shinya Yamanaka at Kyoto University [30]. WD39, another control line, and PB2, a PD patient line with a *PRKN* mutation, were established by Dr. Hideyuki Okano at Keio University [12].

### Generation of TH reporter iPS clones

The control and *PRKN*-mutated patient iPSC lines were cultured on plates coated with iMatrix-511 (Nippi) and expanded in StemFit AK02N medium (Ajinomoto). On day 7 after reseeding, 1 × 10^6^ cells were electroporated at 125 V for 5 ms using a NEPA21 electroporator (NEPAGENE) with 5 µg of Cas9 vectors (Addgene plasmid #60599: a gift from Dr. Akitsu Hotta) [29], sgRNA vectors, and donor vectors into each iPSC line. At day 2 after electroporation, the transfected iPSCs were treated with 0.75 µg/mL of puromycin for 8–9 days. The puromycin-resistant and RFP-positive colonies were manually picked up for PCR screening and expansion. The colonies for expansion were dissociated using TrypLE Select (Thermo Fisher Scientific) and were then cultured with CloneR (StemCell Technologies). The knock-in clones were established following PCR screening.

### PCR and sequencing of TH reporter iPS clones

The identification of knock-in clones was based on the previously reported strategy for screening a reporter iPSC line [31]. PCR primers for the detection of the TH-GFP allele were designed to cover the TH gene and the GFP sequence in the donor vector (Additional file 1: Table S1). PCR primers for the identification of homozygous or heterozygous alleles were designed to cover the sequence before and after the cas9 cleavage sites (Additional file 1: Table S1). The junction of the 5′ homology arm in the TH gene and the T2A-GFP sequence were amplified using primers (Additional file 1: Table S1), and the PCR products (1.2kbp) were sequenced using the Seq reverse primer (Additional file 1: Table S1).

### Differentiation of the TH reporter iPS clones

The generated reporter iPSC lines were differentiated into dopaminergic neurons using previously established the direct neurosphere converting method [14, 22, 32, 33]. For mitochondrial stress, the differentiated cells were treated with 30 μM of the mitochondrial uncoupler, carbonyl cyanide m-chlorophenyl hydrazine (CCCP; Sigma-Aldrich) for 3.5 or 24 h before fixation.

### Immunofluorescence staining and live cell imaging

At day 7–10 of culture after reseeding dissociated neurospheres, differentiated cells were fixed with 4% paraformaldehyde in PBS for 10 min and permeabilized with 0.1% Triton X-100 in PBS for 15 min at room temperature. Cells were then blocked with 2% BSA in PBS for 30 min and stained with primary antibodies for 2 h at room temperature. The following antibodies were used: rabbit anti-TH antibody (PelFreez; 1:400), mouse anti-GFP antibody (MBL; 1:500), mouse anti-β3 tubulin antibody (Sigma-Aldrich; 1:400), mouse anti-Nestin antibody (Sigma-Aldrich; 1:200), and rabbit cleaved caspase-3 antibody (Cell Signaling Technology; 1:400). Cells were then washed three times and stained with Alexa Fluor 488- or 647-conjugated secondary antibodies (Thermo Fisher Scientific; 1:800) and DAPI (Thermo Fisher Scientific; 1:10,000) for 1 h at room temperature. Mitochondrial membrane potential and active mitochondrial mass for live cell imaging were detected by incubation for 30 min with 20 nM tetramethylrhodamine methyl ester (TMRM) (Thermo Fisher Scientific) and 100 nM Mitotracker Deep Red (Thermo Fisher Scientific), respectively. Immunostaining or live cell images were taken using a confocal microscope (Zeiss LSM880).

### Flow cytometry

To detect GFP expression in dopaminergic neurons, differentiated cells were detached from culture plates with Accutase (Nacalai Tesque), and 15,000 cells were analyzed using Guava easyCyte Flow Cytometer (Luminex).

To examine the mitochondrial membrane potential and active mitochondrial mass for flow cytometer, differentiated cells were incubated for 30 min with 5 nM TMRM (Thermo Fisher Scientific) and 10 nM Mitotracker Deep Red (Thermo Fisher Scientific). The cells were detached from culture plates with Accutase (Nacalai Tesque), and 3000 cells were analyzed using FACS Aria IIIu (BD Biosciences).

### Correlative light-electron microscopy

For CLEM analysis, dissociated neurospheres were reseeded on gridded glass-bottom µ-dishes (ibidi) or gridded coverslips (Matsunami) after coating them with poly-l-ornithine and fibronectin. On day 7–10 of culture, cells were fixed in 2% paraformaldehyde, 0.5% glutaraldehyde, and 50 mM sucrose in 0.1 M phosphate buffer. Brightfield and fluorescent images were then taken using a BZ-X710 fluorescence microscope (Keyence). Next, cells were fixed in 2% glutaraldehyde and 50 mM sucrose in 0.1 M phosphate buffer, followed by post fixation with 1% osmium tetroxide. Fixed cells were dehydrated and embedded in Epon812 (Oken Shoji). Ultrathin sections were cut with an ultramicrotome UC6 (Leica) and mounted on glass coverslips. Sections were stained with uranyl acetate and lead citrate, and these coverslips were then coated with carbon using a carbon coater CADE-4T (Meiwa Fosis). Specimens were examined with a scanning electron microscope Helios NanoLab 660 (FEI).

### Experimental design and statistical analysis

The quantitation of fluorescent images was performed for 10 images per iPSC line. The quantitation of electron micrographs was performed in 9–15 cells per iPSC line. GraphPad Prism 8 software was used for statistical analysis. Differences among the groups were evaluated using the unpaired *t*-test or two-way ANOVA with Šidák’s multiple comparisons test, as appropriate. A *P*-value of less than 0.05 was considered significant.

## Results

### Generation of control and *PRKN*-mutated patient TH reporter iPSC lines

To generate TH reporter iPSC lines via the CRISPR/Cas9 system, we designed sgRNA expression vectors. First, we determined a target site with less off-target (Target 1) and a target site near the stop codon (Target 2) for Cas9 nuclease among the exon 12 sequence of the *TH* gene (Fig. 1a). The sgRNA expression vectors were designed by inserting the PCR products, including the Target 1 or 2 sequence, into an RiH vector [29]. Next, we designed the donor vector for insertion into the target sites by homologous recombination (Fig. 1b). To avoid a severe impact on the function of TH by the reporter gene, we chose an HR130PA-1 vector, which is a T2A-based bicistronic expression vector. The 5′ or 3′ homology arms flanking the cleavage site were respectively inserted into each end of the HR130PA-1 vector (T2A-GFP-pA-loxP-EF1α-RFP-T2A-Puro-pA-LoxP-MCS).

The sgRNA, Cas9, and donor vectors were then introduced into the control lines (201B7, WD39) and the PD patient line (PB2). After 8–9 days of puromycin selection, several RFP-positive colonies were observed among the puromycin-resistant colonies in each line, suggesting that the donor sequence was introduced into any site of genomic DNA in the iPS colonies (Fig. 1c). We picked up the RFP-positive colonies and divided each colony into two: one part for DNA isolation and one part for expanding. Purified DNA was amplified using PCR primers for the detection of the TH-GFP allele (Additional file 1: Table S1), to screen the clones that had the GFP sequence at the target site in the *TH* gene. PCR analysis showed that 8 of the 15 RFP-positive colonies that were transfected with the Target 1 sgRNA vector had the GFP sequence at the target site in the *TH* gene (Table 1). In contrast, only 2 of the 20 RFP-positive colonies that were transfected with the Target 2 sgRNA vector had the GFP sequence at the target site in the *TH* gene (Table 1). The low efficiency of the Target 2 sgRNA is likely caused by its high number of off-targets relative to Target 1. To determine whether the clones were homozygous or heterozygous for the TH-GFP allele, clones with the TH-GFP allele were expanded and further analyzed for unedited TH allele using PCR. As a result, a B7 knock-in clone (201B7 T1-3) and three of the four PB2 knock-in clones (PB2 T1-1, -2, and -4) carried homozygous TH-GFP alleles (Fig. 1d). In contrast, all of the WD39 knock-in clones carried heterozygous TH-GFP alleles (Fig. 1d). Sanger sequencing demonstrated that the T2A-GFP sequence was correctly inserted at the cleavage site in all of four knock-in iPSC lines used in this study: a control homozygous line (B7 T1-3), a control heterozygous line (WD39 T1-2), and two patient homozygous lines (PB2 T1-1 and -4) (Fig. 1e). Thus, these data demonstrate the successful generation of control and *PRKN*-mutated patient TH reporter iPSC (TH-GFP iPSC) lines.

**Table 1:** Screening of control and *PRKN*-mutated TH-GFP iPSC lines by PCR analysis for TH-GFP allele

Original iPSC line	Target site	No. of RFP-positive colonies	No. of colonies with TH-GFP allele
Control (201B7)	Target 1	6	1
	Target 2	5	0
Control (WD39)	Target 1	3	3
	Target 2	6	2
PRKN (PB2)	Target 1	6	4
	Target 2	9	0

### Differentiation of the TH-GFP iPSC lines into dopaminergic neurons

**Fig. 2:**
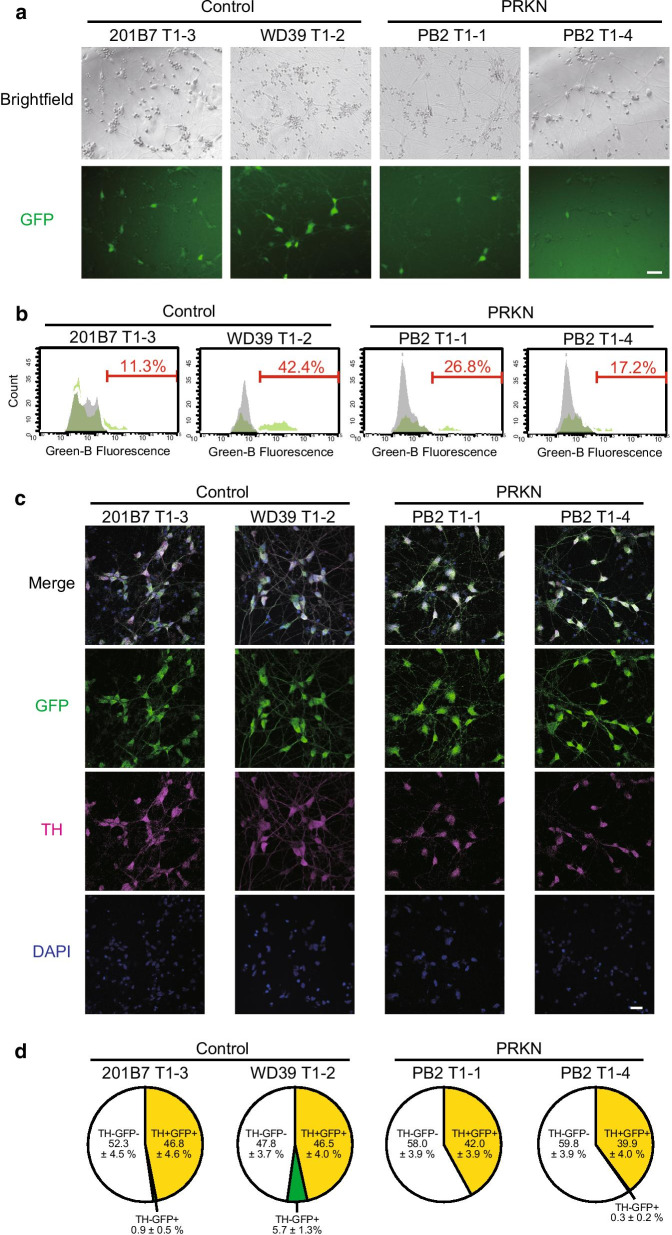
Characterization of dopaminergic neurons differentiated from TH-GFP iPSC lines. **a** The brightfield and fluorescent images at day 7 after reseeding the neurospheres demonstrated GFP expression in some of the differentiated neurons derived from TH-GFP iPSC lines. “PRKN” represents *PRKN*-mutated patient. Scale bar, 50 µm. **b** Flow cytometric analysis showed GFP-positive fractions in the TH-GFP iPSC-derived neurons against the original iPSC-derived neurons at day 9 after reseeding the neurospheres. “PRKN” represents *PRKN*-mutated patient. **c** Immunofluorescence staining for GFP and TH confirmed similar expression patterns among TH-GFP iPSC-derived neurons. “PRKN” represents *PRKN*-mutated patient. Scale bar, 20 µm. **d** Pie graphs showing quantitative analysis of the proportion of TH+GFP+ (yellow areas), TH−GFP+ (green areas), TH+GFP− , or TH−GFP− (white areas) cells in the total DAPI+ cells from the immunofluorescence images (n = 10 per iPSC line) in (**c**). There were no TH+GFP− cells derived from any TH-GFP iPSC lines. “PRKN” represents *PRKN*-mutated patient. Values are shown as the mean ± SEM

To confirm the expression of the TH-GFP gene, we differentiated the generated TH-GFP iPSC lines into dopaminergic neurons using previously reported the direct neurosphere converting method [14, 33]. On day 7 after reseeding the neurospheres, GFP expression was detected in differentiated neurons derived from all of four TH-GFP iPSC lines (Fig. 2a). Flow cytometric analysis demonstrated that 11.3–42.4% GFP-positive fractions existed in TH-GFP iPSC-derived neurons on day 9 after reseeding the neurospheres (Fig. 2b). In the WD39 T1-2 neurons, which carried heterozygous TH-GFP alleles, GFP expression levels were comparable to those of other homozygous reporter lines (Fig. 2b).

To examine the specificity of the TH reporter, we stained the TH-GFP iPSC-derived neurons with anti-TH and anti-GFP antibodies and confirmed a strong colocalization of TH and GFP (Fig. 2c). We measured the proportion of TH/GFP double-positive, TH or GFP single-positive, and TH/GFP double-negative cells in the immunofluorescence images. TH-positive/GFP-negative and TH-negative/GFP-positive cells were hardly detected in the immunofluorescence images, suggesting that the generated TH-GFP iPSC lines had high specificity for the TH reporter (Fig. 2c, d). The proportion (39.9–46.8%) of TH-positive dopaminergic neurons in the total differentiated cells was comparable to that of the original iPSC lines [13] (Fig. 2d). However, the proportions of GFP-positive neurons were lower when analyzed in flowcytometry (11.3–42.4%) (Fig. 2b) compared with the quantitation of TH/GFP-positive dopaminergic neurons in immunostaining (39.9–46.8%) (Fig. 2d). This discrepancy probably resulted from the higher detection of TH/GFP by immunostaining in neurons expressing low levels of TH/GFP.

**Fig. 3:**
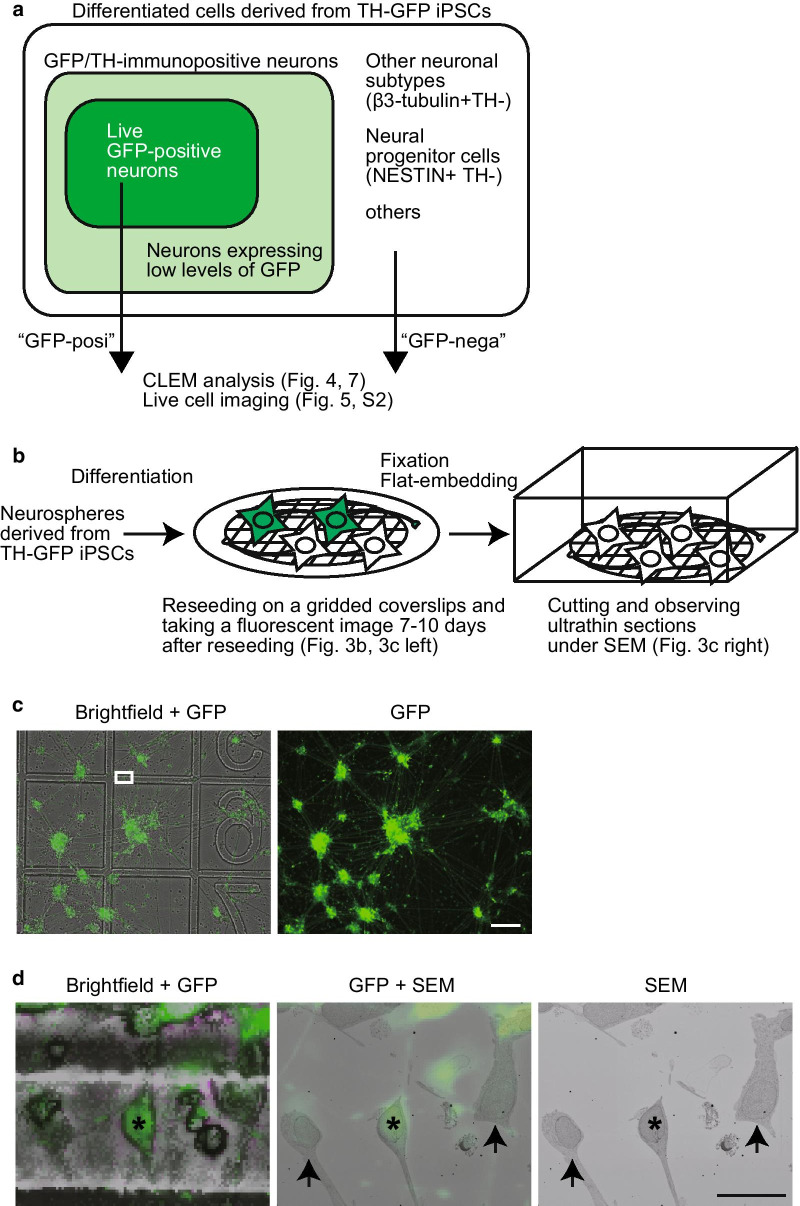
CLEM analysis of GFP-positive and GFP-negative cells derived from TH-GFP iPSCs. **a** Diagram representing the comparative analysis of GFP-positive and -negative cells in CLEM analysis (Figs. 4 and 7) and live cell imaging (Fig. 5, Additional file 3: Figure S2). Neurons expressing high levels of GFP (green in the diagram) were defined as “GFP-positive” cells. Cells expressing no levels of GFP and with a neuronal shape, which were probably other neuronal subtypes or neural progenitors (white in the diagram), were defined as “GFP-negative” cells. Neurons expressing low levels of GFP (light green in the diagram) were not used in either CLEM analysis or live cell imaging. **b** Scheme of the CLEM procedure. Neurospheres differentiated from TH-GFP iPSCs were dissociated and reseeded on the gridded coverslips. After 7–10 days of culture, the differentiated neurons consisted of a mixture of GFP-positive (green) dopaminergic neurons and GFP-negative (white) non-dopaminergic neurons. Brightfield and fluorescent images were taken of cells on the gridded coverslips, and the cells were further fixed and flat-embedded for electron microscopic analysis. The cells of interest in the ultrathin sections were identified with help from the grid patterns and cell shapes using an SEM. **c** The GFP (right) and merged (brightfield + GFP, left) images of TH-GFP iPSC-derived cells on the gridded coverslips. The white box indicates the origin of the enlarged image (**c**, left). Scale bar, 100 µm. **d** The brightfield and GFP (left), GFP and SEM (center), and SEM (right) images. Asterisks and arrows indicate GFP-positive and -negative cells, respectively. Scale bar, 20 µm

To characterize the TH/GFP-double negative cells, we stained the TH-GFP iPSC-derived cells with anti-β3-tubulin and anti-Nestin antibodies. Both β3-tubulin-positive/TH-negative cells and Nestin-positive/TH-negative cells existed in the all lines of TH-GFP iPSC-derived neurons (Additional file 2: Figure S1). This result suggests that the TH/GFP-negative cell population contains other neuronal subtypes and neural progenitor cells (Additional file 2: Figure S1, Fig. 3a). Together, these findings indicate that our generated control and *PRKN*-mutated patient TH-GFP iPSC lines can be used for dopaminergic neuron-specific analyses of control and *PRKN*-mutated patient.

### CLEM analysis of mitochondrial morphology in GFP-positive and/or GFP-negative cells derived from TH-GFP iPSCs

The death of dopaminergic neurons in *PRKN*-related PD is assumed to be caused by the accumulation of damaged mitochondria [4, 5]. To investigate the mitochondrial morphology specific to dopaminergic neurons under normal conditions, we planned to compare the ultrastructure of dopaminergic neurons with non-dopaminergic neurons. For this purpose, we used a CLEM strategy, which allows to gain not only the location of dopaminergic and non-dopaminergic neurons but also the fine ultrastructural information, because the strong fixation for electron microscopy is performed after fluorescent images are taken (Fig. 3b). The TH-GFP iPSC-derived neurons were seeded on gridded coverslips, and brightfield and fluorescent images were taken before the cells were fixed strongly and processed for electron microscopy (Fig. 3b). For the CLEM analyses, we selected cells with either strong GFP expression or no GFP expression at all (Fig. 3a), because in the fluorescent imaging, neurons with weak expression of TH /GFP were difficult to detect. We marked 10–15 GFP-positive and -negative cells per TH-GFP cell line on the brightfield and fluorescent images (Fig. 3c). We then searched for the marked cells on the ultrathin sections, with help from grid patterns and cell shapes, under a scanning electron microscope (Fig. 3d).

**Fig. 4:**
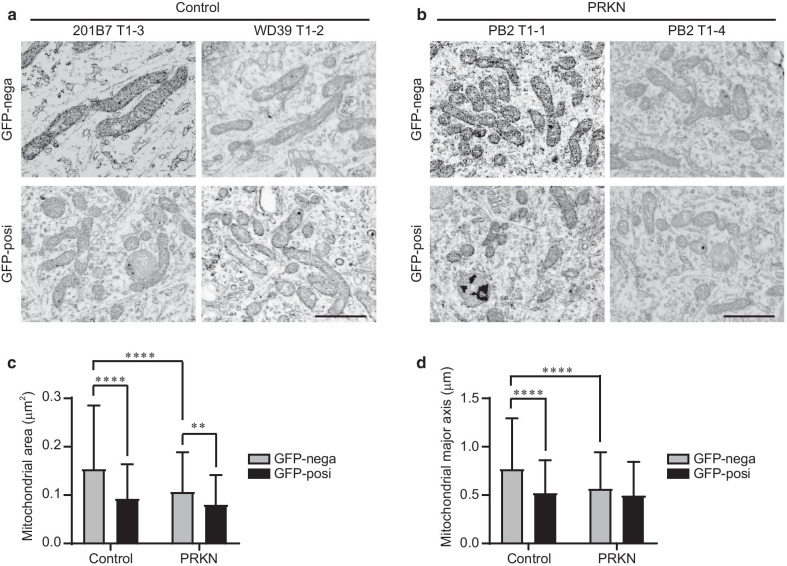
Cell type-specific mitochondrial morphology in dopaminergic neurons derived from TH-GFP iPSCs. **a** Representative SEM images of GFP-negative non-dopaminergic neurons (top) and GFP-positive dopaminergic neurons (bottom) derived from control TH-GFP iPSCs. Scale bar, 1 µm. **b** Representative SEM images of GFP-negative non-dopaminergic neurons (top) and GFP-positive dopaminergic neurons (bottom) derived from *PRKN*-mutated TH-GFP iPSCs. “PRKN” represents *PRKN*-mutated patient. Scale bar, 1 µm. **c** Quantitative analysis of the mitochondrial area from the SEM images of GFP-negative and -positive cells derived from control and *PRKN*-mutated TH-GFP iPSCs. “PRKN” represents *PRKN*-mutated patient. Mitochondrial area (control GFP-nega; n = 199, control GFP-posi; n = 198, PRKN GFP-nega; n = 215, PRKN GFP-posi; n = 200) was measured using ImageJ. Values are shown as the mean ± SD. Statistical significance was evaluated using the two-way ANOVA with Šidák’s multiple comparisons test. **P < 0.01, ****P < 0.0001. There was no significant difference in the mitochondrial area between control and *PRKN*-mutated GFP-positive cells (*P* = 0.298). **d** Quantitative analysis of the mitochondrial major axis from the SEM images of GFP-negative and -positive cells derived from control and *PRKN*-mutated TH-GFP iPSCs. “PRKN” represents *PRKN*-mutated patient. The mitochondrial major axis (control GFP-nega; n = 199, control GFP-posi; n = 198, PRKN GFP-nega; n = 215, PRKN GFP-posi; n = 200) was measured using ImageJ. Values are shown as the mean ± SD. Statistical significance was evaluated using the two-way ANOVA with Šidák’s multiple comparisons test. ****P < 0.0001. There was no significant difference in the mitochondrial major axis between the GFP-positive and -negative *PRKN*-mutated cells (*P* = 0.1651). There was no significant difference in the mitochondrial major axis between control and *PRKN*-mutated GFP-positive cells (*P* = 0.7795)

Almost all of the marked cells were found and observed for mitochondrial morphology in the ultrathin sections. Under normal conditions mitochondrial structural changes were not found in GFP-positive and -negative cells derived from control and *PRKN*-mutated iPSC lines, as far as we observed using CLEM analysis. On the other hand, CLEM analysis revealed that mitochondrial size tended to be smaller in the GFP-positive dopaminergic neurons compared with the GFP-negative non-dopaminergic neurons, in all of the TH-GFP lines (Fig. 4a, b). To confirm this finding, we measured the area and major axis of each mitochondrion in the electron microscope images. Quantitation of the mitochondrial area showed that mitochondria in GFP-positive dopaminergic neurons were significantly smaller than in GFP-negative non-dopaminergic neurons in both the control and *PRKN*-mutated lines (Fig. 4c control, *P* < 0.0001; Fig. 4c PRKN, *P* = 0.0064). The mitochondrial major axes in GFP-positive dopaminergic neurons were also significantly shorter than in GFP-negative non-dopaminergic neurons in control lines (Fig. 4d control, *P* < 0.0001). In *PRKN*-mutated lines, the mitochondrial major axis in GFP-positive dopaminergic neurons tended to be shorter than that in GFP-negative non-dopaminergic neurons, but this difference was not statistically significant (Fig. 4d PRKN, *P* = 0.1651). There were significant differences of mitochondrial area and major axis in non-dopaminergic neurons between control and *PRKN*-mutated lines (Fig. 4c, *P* < 0.0001; Fig. 4d, *P* < 0.0001), suggesting that the loss of Parkin altered the mitochondrial morphology in non-dopaminergic neurons. Together, these results indicate that the iPSC-derived dopaminergic neurons had smaller mitochondria than the non-dopaminergic neurons in both the control and *PRKN*-mutated lines.

### Mitochondrial membrane potential in GFP-positive and -negative cells derived from TH-GFP iPSCs

**Fig. 5:**
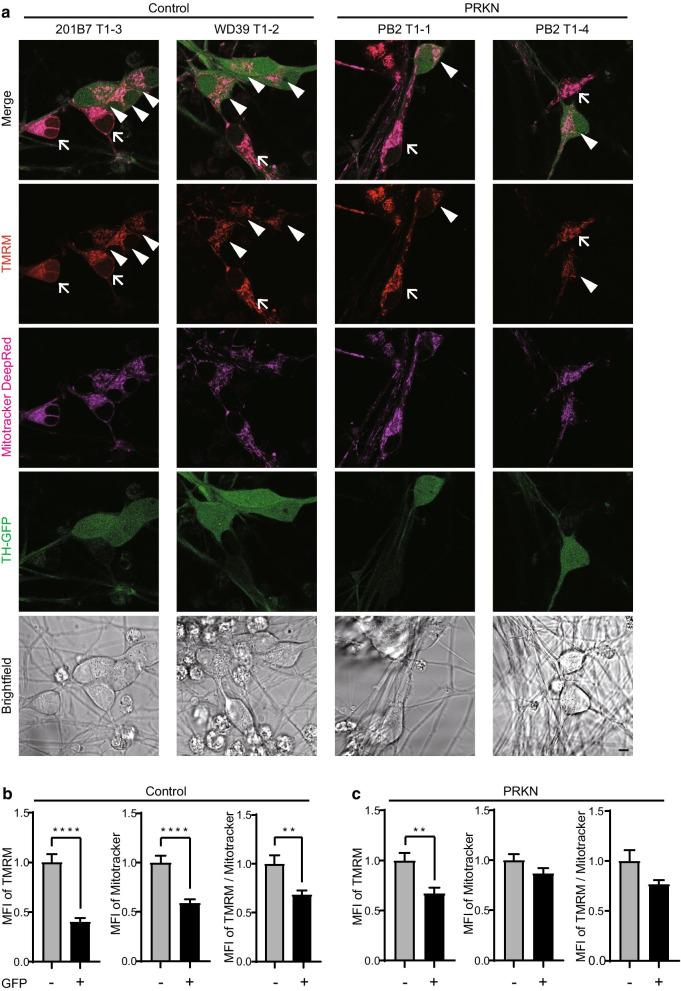
Live cell imaging of mitochondrial membrane potential in dopaminergic neurons derived from TH-GFP iPSCs. **a** Live cell imaging of dopaminergic and non-dopaminergic neurons derived from control and *PRKN*-mutated TH-GFP iPSCs. Arrowheads indicate weak TMRM signals in GFP-positive dopaminergic neurons. Arrows indicate strong TMRM signals in GFP-negative non-dopaminergic neurons. “PRKN” represents *PRKN*-mutated patient. Scale bar, 5 µm. **b** Quantitative analysis of the MFI of TMRM (left), Mitotracker DeepRed (center), and TMRM normalized to Mitotracker DeepRed (right) in control GFP-positive cells relative to control GFP-negative cells in fluorescence images (n = 20 images). The MFI was measured using the surrounding cytoplasmic area in ZEN software. Values are shown as the mean ± SEM. Statistical significance was evaluated using the unpaired two-tailed *t*-test. **P < 0.01, ****P < 0.0001. **c** Quantitative analysis of the MFI of TMRM (left), Mitotracker DeepRed (center), and TMRM normalized with Mitotracker DeepRed (right) in *PRKN*-mutated GFP-positive cells relative to *PRKN*-mutated GFP-negative cells in fluorescence images (n = 20 images). “PRKN” represents *PRKN*-mutated patient. Values are shown as the mean ± SEM. Statistical significance was evaluated using the unpaired two-tailed *t*-test. **P < 0.01. There were no significant differences in the MFI of Mitotracker and TMRM/Mitotracker between GFP-positive and -negative cells (b″; *P* = 0.1163, b‴; *P* = 0.0551)

To further investigate whether mitochondrial function in dopaminergic neurons is lower than in non-dopaminergic neurons, we compared mitochondrial membrane potentials in living GFP-positive dopaminergic neurons and GFP-negative non-dopaminergic neurons using TMRM, a fluorescent probe for monitoring the mitochondrial membrane potential. Live cell imaging showed that the fluorescent signal of TMRM in GFP-positive dopaminergic neurons was remarkably lower than in GFP-negative non-dopaminergic neurons in both the control and *PRKN*-mutated lines (Fig. 5a). Quantitation of the mean fluorescent intensity (MFI) of TMRM confirmed that the mitochondrial membrane potential per cell in dopaminergic neurons was significantly lower than in non-dopaminergic neurons (Fig. 5b left, *P* < 0.0001; Fig. 5c left, *P* = 0.0015).

Next, we quantified the MFI of Mitotracker Deep Red, which is a fluorescent dye of mitochondria. We then normalized the MFI of TMRM to the MFI of Mitotracker Deep Red to examine the membrane potential per mitochondria (Fig. 5b center, 5b right, 5c center, and 5c right). The mitochondrial membrane potential normalized to the mitochondrial mass was also significantly lower in dopaminergic neurons than in non-dopaminergic neurons in the control lines (Fig. 5b right, *P* = 0.0023). In the *PRKN*-mutated lines, the mitochondrial membrane potential normalized to the mitochondrial mass tended to be lower in dopaminergic neurons than in non-dopaminergic neurons, but this difference was not significant (Fig. 5c right, *P* = 0.0551).

To further confirm the mitochondrial membrane potentials in dopaminergic and non-dopaminergic neurons, we stained the differentiated cells with TMRM and Mitotracker Deep Red for flow cytometric analysis. Flow cytometric analysis showed that 6.5%-28.6% GFP-positive fractions existed in the differentiated cells derived from each TH-GFP iPSC lines (Additional file 3: Figure S2a). Consistent with the results from live cell imaging, TMRM-GFP cytograms showed that GFP-positive populations had the lower TMRM signal compared to GFP-negative populations in the control lines (Additional file 3: Figure S2b). Quantitation of the MFI of TMRM demonstrated that the mitochondrial membrane potentials per cell/mitochondria in GFP-positive dopaminergic neurons were significantly lower than that in GFP-negative non-dopaminergic neurons in the control lines (Additional file 3: Figure S2c control left, *P* = 0.0169; Figure S2c control right, *P* = 0.0315). In the *PRKN*-mutated lines, the mitochondrial membrane potentials per cell/mitochondria in GFP-positive dopaminergic neurons tended to be lower than that in GFP-negative non-dopaminergic neurons, although these differences were not significant (Additional file 3: Figure S2c PRKN left, *P* = 0.5567; Figure S2c PRKN right, *P* = 0.1382). Contrary to the results from live cell imaging, the mitochondrial mass in *PRKN*-mutated dopaminergic neurons was significantly higher than that in *PRKN*-mutated non-dopaminergic neurons (Additional file 3: Figure S2c PRKN center, *P* = 0.0438).

These results indicate that iPSC-derived dopaminergic neurons have lower mitochondrial membrane potentials not only at the individual mitochondrial level but also at the cellular level. Nevertheless, the differences in mitochondrial function and size between dopaminergic and non-dopaminergic neurons in the control lines were more significant than in the *PRKN*-mutated lines. This result suggests that the smaller size and lower function of mitochondria in the dopaminergic neurons are probably not the result of the *PRKN* mutation, but are rather characteristic of dopaminergic neurons themselves.

### Cell death of dopaminergic neurons derived from *PRKN*-mutated patient TH-GFP iPSC lines under CCCP

**Fig. 6:**
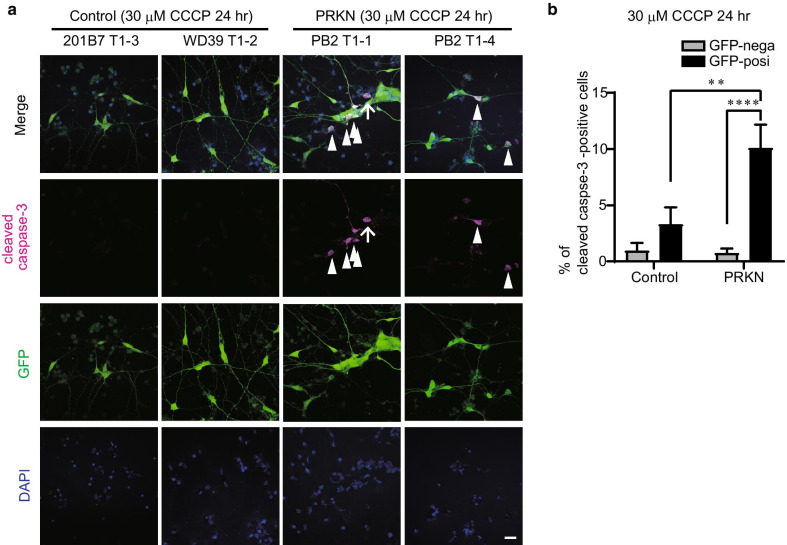
Pathogenicity of dopaminergic neurons differentiated from *PRKN*-mutated TH-GFP iPSC lines. **a** Immunofluorescence staining of cleaved caspase-3 and GFP in TH-GFP iPSC-derived neurons under CCCP treatment for 24 h indicated that *PRKN*-mutated dopaminergic neurons were susceptible to cell death under oxidative stress. Arrowheads represent cleaved caspase-3/GFP double-positive *PRKN*-mutated cells. The arrow represents a cleaved caspase-3 single-positive *PRKN*-mutated cell. “PRKN” represents *PRKN*-mutated patient. Scale bar, 20 µm. **b** Quantitative analysis of the proportion of cleaved caspase-3-positive cells in GFP-positive dopaminergic neurons or GFP-negative non-dopaminergic neurons, determined from immunofluorescence images (n = 10 images per iPSC line) as in (**a**). “PRKN” represents *PRKN*-mutated patient. Values are shown as the mean ± SEM. Statistical significance was evaluated using the two-way ANOVA with Šidák’s multiple comparisons test. **P < 0.01, ****P < 0.0001. There was no significant difference in the proportion of cleaved caspase-3-positive cells between control and *PRKN*-mutated GFP-negative cells (*P* = 0.9901). There was also no significant difference in the proportion of cleaved caspase-3-positive cells between GFP-negative and -positive cells in the control lines (*P* = 0.3822)

A previous study has reported that *PRKN*-mutated dopaminergic neurons show increased cell death compared with control lines under CCCP treatment for 24 h [13]. To evaluate the cell death of dopaminergic neurons in the generated control and *PRKN*-mutated TH-GFP iPSC lines, the TH-GFP iPSC-derived cells were treated with CCCP for 24 h and then stained with the antibody against cleaved caspase-3, which is a marker of apoptosis. There were significantly increased numbers of cleaved caspase-3 -positive cells in the *PRKN*-mutated dopaminergic neurons compared with both the control dopaminergic neurons (Fig. 6a, b; *P* = 0.0011) and the *PRKN*-mutated non-dopaminergic neurons (Fig. 6a, b; *P* < 0.0001). These results suggest that the TH-GFP *PRKN*-mutated patient iPSC line replicates the typical pathological phenotype of PD with *PRKN* mutations: the preferential loss of dopaminergic neurons.

### Electron microscopic analysis of dopaminergic neurons derived from *PRKN*-mutated TH-GFP iPSCs under CCCP treatment

**Fig. 7:**
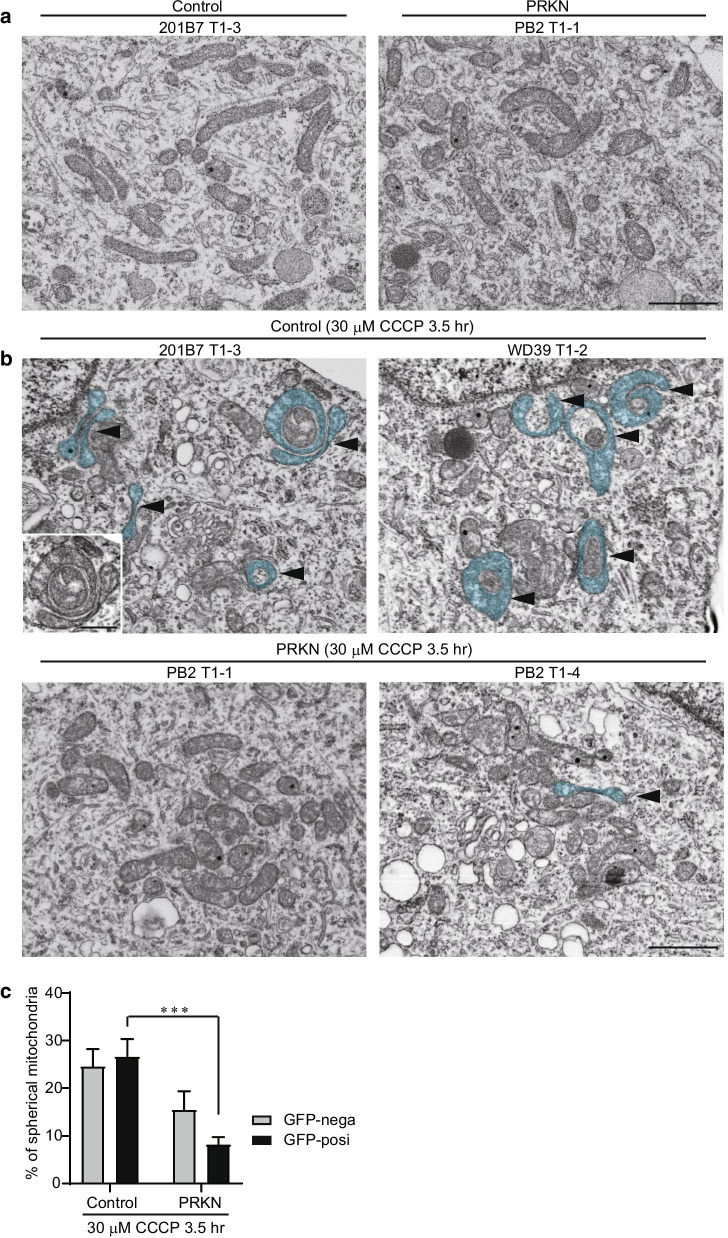
Spheroidal formation of mitochondria in iPSC-derived dopaminergic neurons under CCCP treatment. **a** SEM images of GFP-positive dopaminergic neurons derived from control and *PRKN*-mutated TH-GFP iPSC lines under normal conditions. “PRKN” represents *PRKN*-mutated patient. No spherical mitochondria of total mitochondria (control GFP-nega; n = 745, control GFP-posi; n = 1427, PRKN GFP-nega; n = 811, PRKN GFP-posi; n = 1178) was observed in the SEM images of differentiated cells. Scale bar, 1 µm. **b** SEM images of GFP-positive dopaminergic neurons derived from control and *PRKN*-mutated TH-GFP iPSC lines under CCCP treatment for 3.5 h. Mitochondrial structures painted cyan and indicated by arrowheads represent spherical mitochondria. “PRKN” represents *PRKN*-mutated patient. Scale bar, 1 µm. The inset shows an image of one of the spherical mitochondria without painting. Scale bar of the inset, 500 nm. **c** Quantitative analysis of the percentage of spherical mitochondria in total mitochondria (control GFP-nega; n = 562, control GFP-posi; n = 850, PRKN GFP-nega; n = 736, PRKN GFP-posi; n = 774) from the SEM images of differentiated cells, as in (**a**). “PRKN” represents *PRKN*-mutated patient. Values are shown as the mean ± SEM. Statistical significance was evaluated using the two-way ANOVA with Šidák’s multiple comparisons test. ***P < 0.001. There was no significant difference in the percentage of spherical mitochondria between control and *PRKN*-mutated GFP-negative cells (*P* = 0.1038). There was also no significant difference in the percentage of spherical mitochondria between GFP-negative and GFP-positive cells (control; *P* = 0.8825, PRKN; *P* = 0.2222)

As shown above, we were unable to identify any differences in mitochondria between the *PRKN*-mutated and control lines under normal conditions using CLEM and live cell imaging. CLEM analysis demonstrated that normal condition induced no mitochondrial structural change in both control and *PRKN*-mutated lines (Fig. 7a). We, therefore, treated the TH-GFP iPSC-derived cells with CCCP for 3.5 h to detect the initial changes in mitochondrial structure before cell death.

We first confirmed that CCCP treatment for 3.5 h reduced the mitochondrial membrane potential in control TH-GFP iPSC-derived cells using live cell imaging with TMRM (Additional file 4: Figure S3). Next, we observed mitochondrial ultrastructure in the CCCP-treated dopaminergic neurons using CLEM. CLEM analysis revealed that CCCP treatment in control GFP-positive dopaminergic neurons induced the transformation of mitochondria into ring-shaped (donut-shaped) structures (Fig. 7b control). The mitochondrial morphological changes from tubular forms to ring-shaped forms have previously reported in MEF and HeLa cells under CCCP treatment, and the mitochondria with the characteristic ring-shaped structures are called spherical mitochondria [17, 18, 34, 35]. In contrast, few spherical mitochondria were observed in *PRKN*-mutated GFP-positive dopaminergic neurons (Fig. 7b PRKN). By calculating the ratio of spherical mitochondria, we demonstrated that the formation of spheroid-shaped mitochondria was significantly reduced in *PRKN*-mutated dopaminergic neurons (Fig. 7c; *P* = 0.0003). There was no significant difference in the ratio of spherical mitochondria between control and *PRKN*-mutated lines in non-dopaminergic neurons (Fig. 7c; *P* = 0.1038). The finding indicates that CCCP treatment induces significant differences in mitochondrial structure between control and *PRKN*-mutated dopaminergic neurons.

## Discussion

In the present study, we first established TH-GFP iPSC lines from not only controls but also a patient with a *PRKN* mutation and first analyzed these cells using live cell imaging and CLEM to evaluate the structure and function of mitochondria specifically in dopaminergic neurons. Several studies have reported the establishment of TH reporter iPSC lines using gene editing technology for transcriptome profiling or the electrical properties of dopaminergic neurons [23–25]. Immunohistochemistry confirmed that the specificity of our TH-GFP iPSC lines was sufficient for the identification of dopaminergic neurons, similar to that of other knock-in reporters using genome editing technology. There was a small variation in the proportion of GFP-positive cells, possibly due to the difference in the cell line-specific gene expression pattern. The *PRKN*-mutated TH-GFP iPSC lines replicated the preferential death of dopaminergic neurons caused by CCCP treatment for 24 h. This result indicates that our established control and *PRKN*-mutated TH-GFP iPSC lines are useful for analyzing the specific phenotype of dopaminergic neurons in patients with *PRKN* mutations. We aimed to use the TH-GFP iPSC lines for CLEM analysis of mitochondrial ultrastructure, because mitochondrial ultrastructure has not been clearly examined in control and *PRKN*-mutated patient dopaminergic neurons in previous studies.

CLEM analysis and live cell imaging under normal conditions demonstrated that dopaminergic neurons had smaller and less functional mitochondria compared with non-dopaminergic neurons in both the control and *PRKN*-mutated lines. Our results regarding mitochondrial size in dopaminergic neurons are consistent with those of a previous study, which reported significantly smaller mitochondria in substantia nigra dopaminergic neurons compared with non-dopaminergic neurons in the mouse brain [36]. Our data present the first in vitro evidence of smaller mitochondria in human iPSC-derived dopaminergic neurons.

Our quantitation of mitochondrial size in CLEM also provided evidence that the mean mitochondrial size in cells derived from the *PRKN*-mutated patient tended to be smaller than that of control cells in both dopaminergic and non-dopaminergic neurons. Similarly, a previous study of iPSC-derived neurons using electron microscopy showed that control neurons have long cylindrical mitochondria compared with *PRKN*-mutated neurons, although mitochondrial ultrastructure was not specifically analyzed in dopaminergic neurons [12]. Together, these results suggest that mitochondrial size in both dopaminergic and non-dopaminergic neurons may be affected by *PRKN* mutations. However, the present study showed that the *PRKN* mutation had little effect on mitochondrial size when comparing dopaminergic neurons between control and *PRKN*-mutated lines. This result indicates the importance of using labeled dopaminergic neurons for studying dopaminergic neuron-specific phenotypes in PD.

Live cell imaging and flow cytometric analysis of TMRM-stained cells demonstrated that the mitochondrial membrane potential in dopaminergic neuron was markedly lower than that in non-dopaminergic neuron at least in the control lines. This finding implies that mitochondrial function in dopaminergic neurons is correlated with mitochondrial morphology. This observed correlation between mitochondrial morphology and function is supported by many previously published studies. For example, nuclear reprogramming can change oxidative differentiated cells with tubular elongated mitochondria into glycolytic pluripotent stem cells with round-shaped mitochondria [37, 38]. Furthermore, several reports have demonstrated that artificial mitochondrial fragmentation results in impaired mitochondrial function and increased cell death [39–42].

On the other hand, a previous study reported that dopaminergic neurons of the substantia nigra pars compacta have a higher basal rate of mitochondrial oxidative phosphorylation and more complex axons than those of the ventral tegmental area in the mouse [43]. In the study, mitochondrial function in dopaminergic neurons was measured in the whole cell using an extracellular flux analyzer. In our flow cytometric analysis, the decline of the mitochondrial membrane potential in the dopaminergic neurons was milder than that in the soma in live cell imaging. The decline of the mitochondrial membrane potential in the whole cell was probably complemented with a large number of axonal mitochondria in the dopaminergic neurons. In fact, the decline rate of the mitochondrial membrane potential normalized by the mitochondrial mass of the dopaminergic neurons in flow cytometry was similar to that in live cell imaging. Our findings of the small size and low function of mitochondria in the dopaminergic neurons provide a dopaminergic neuron-specific characterization of mitochondria, and would also elucidate one of the possible causes of the high vulnerability of dopaminergic neurons in both familial and sporadic PD.

In the present study, there was no difference in the mitochondrial structure of dopaminergic neurons between control and *PRKN*-mutated lines under normal conditions using CLEM. However, CCCP treatment induced significant differences in mitochondrial structure and cell death between control and *PRKN*-mutated dopaminergic neurons. CCCP treatment for 3.5 h induced the formation of spheroid-shaped mitochondria in control dopaminergic neurons; in contrast, the CCCP-induced formation of spheroid-shaped mitochondria was significantly impaired in *PRKN*-mutated dopaminergic neurons. Spheroid-shaped mitochondria as a result of mitochondrial uncouplers have been observed in MEF, HEK-293, and HeLa cells, and this spheroid shape is considered to be the morphology of uncoupled mitochondria ready for degradation by lysosomes [17, 18, 34, 35]. CCCP treatment is accompanied by the colocalization of mitochondria with lysosomes, irrespective of mitophagy, suggesting that the formation of spheroid-shaped mitochondria may be a novel mechanism of mitochondrial quality control [18]. The present study is the first report of the formation of spheroid-shaped mitochondria in human iPSC-derived dopaminergic neurons. A recent study suggested that spheroid-shaped mitochondrial morphology represents the more stable form of uncoupled mitochondria, and that spheroid-shaped mitochondria undergo mitochondrial degradation [35]. Additionally, previous studies of *PRKN*-mutated patient iPSC-derived neurons have reported that CCCP treatment for 48 h induces the accumulation of damaged mitochondria [12, 14]. Thus, it is hypothesized that defects in the formation of spheroid-shaped mitochondria may disturb the degradation of damaged mitochondria in *PRKN*-mutated dopaminergic neurons, resulting in the subsequent cell death of *PRKN*-mutated dopaminergic neurons.

A study by Ding et al. demonstrated that mitochondrial spheroid formation is increased by the knockdown of Parkin in HEK-293 cells [17]. Our study indicated that in dopaminergic neurons the formation of spherical mitochondria was significantly decreased in *PRKN*-mutated lines when compared with control lines. On the other hand, in non-dopaminergic neurons, there was no significant difference in the ratio of the formation of spherical mitochondria between control and *PRKN*-mutated lines. These differences in the effect of Parkin deficiency on the formation of spherical mitochondria possibly results from the difference in cell types. Taken together, our CLEM analysis of TH-GFP iPSC-derived dopaminergic neurons provided evidence of mitochondrial structural changes before cell death. Furthermore, our study suggests the possibility that defect in mitochondrial spheroid formation contributes to the accumulation of damaged mitochondria and the death of dopaminergic neurons in patients with *PRKN* mutations. However, the limitation in this study is the small number of TH-GFP iPSC lines derived from two healthy subjects and a patient. Further studies with increased number of TH-GFP iPSC lines and Parkin knockdown/knockout studies using our control iPSC lines are expected to resolve any controversy about the involvement of Parkin expression in mitochondrial spheroid formation.

In conclusion, the current study demonstrated the establishment of control and *PRKN*-mutated patient TH-GFP iPSC lines for mitochondrial ultrastructural analysis specific to *PRKN*-mutated dopaminergic neurons. CLEM analysis of the TH-GFP iPSC-derived cells revealed the existence of mitochondrial morphology specific to dopaminergic neurons, as well as the insufficient spheroid formation of mitochondria under CCCP treatment in *PRKN*-mutated dopaminergic neurons. Although the direct relationships between the mitochondrial morphology and the cell death in *PRKN*-mutated dopaminergic neurons have not been clarified in the present study, our in vitro model newly resulted in the discovery of differences in mitochondrial ultrastructural changes between control and *PRKN*-mutated patient under mitochondrial stress. Future studies using our model would reveal the association between defect in mitochondrial spheroid formation and the accumulation of damaged mitochondria and the death of dopaminergic neurons in patients with *PRKN* mutations. In addition, our workflow—using CLEM analysis of TH-GFP iPSC lines—is also a strong tool for evaluating mitochondrial-derived vesicles, the ER-mitochondria interface, or other organelle impairments in *PRKN*-mutated patient dopaminergic neurons specifically, under normal and/or pathological conditions, which will help to elucidate the processes leading to the preferential loss of dopaminergic neurons in patients with *PRKN* mutations.

## Supplementary Information

**Additional file 1: Table S1.** List of the primers used in this study.

**Additional file 2: Figure S1.** Characterization of non-dopaminergic neurons differentiated from TH-GFP iPSC lines. Immunofluorescence staining for TH, β3-tubulin, and Nestin identified other neural subtypes (β3-tubulin + TH-) and neural progenitor cells (Nestin + TH-) among the non-dopaminergic cells derived from TH-GFP iPSC lines. Arrowheads indicate β3-tubulin + TH- or Nestin + TH- cells. “PRKN” represents *PRKN*-mutated patient. Scale bar, 20 µm.

**Additional file 3: Figure S2.** Flow cytometric analysis of mitochondrial membrane potential in dopaminergic neurons derived from TH-GFP iPSCs. (a) Histograms showed GFP-negative and GFP-positive populations in the TH-GFP iPSC-derived differentiated cells. Data were obtained from three independent experiments. “PRKN” represents *PRKN*-mutated patient. Values are shown as the mean ± SEM. (b) TMRM-GFP cytograms (top) represented the intensity of TMRM signal in GFP-negative and -positive populations. Mitotracker DeepRed-GFP cytograms (bottom) represented the intensity of Mitotracker DeepRed signal in GFP-positive and -positive populations. “PRKN” represents *PRKN*-mutated patient. (c) Quantitative analysis of the MFI of TMRM (left), Mitotracker DeepRed (center), and TMRM normalized with Mitotracker DeepRed (right) in GFP-positive cells relative to GFP-negative cells in the control and *PRKN*-mutated lines. Data were obtained from three independent experiments. “PRKN” represents *PRKN*-mutated patient. Values are shown as the mean ± SEM. Statistical significance was evaluated using the unpaired two-tailed *t*-test. *P < 0.05. There was no significant difference in the MFI of Mitotracker between control GFP-positive and -negative cells (control center; *P* = 0.3502). There were no significant differences in the MFI of TMRM and TMRM/Mitotracker between *PRKN*-mutated GFP-positive and -negative cells (PRKN left; *P* = 0.5567, PRKN right; *P* = 0.1382).

**Additional file 4: Figure S3.** Mitochondrial membrane potential in iPSC-derived cells under CCCP treatment. Mitochondrial membrane potential in dopaminergic and non-dopaminergic neurons derived from control TH-GFP iPSCs under normal conditions and CCCP treatment. CCCP treatment for 3.5 h rapidly attenuated the fluorescence of TMRM. Scale bar, 5 µm.

## Data Availability

The datasets used and/or analysed during the current study are available from the corresponding author on reasonable request.
