# Supplementary figures and images for: Establishment of an in vitro model for analyzing mitochondrial ultrastructure in PRKN-mutated patient iPSC-derived dopaminergic neurons
Source: Mol Brain. 2021 Mar 23;14:58. doi: 10.1186/s13041-021-00771-0 (PMC7986497; doi:10.1186/s13041-021-00771-0)

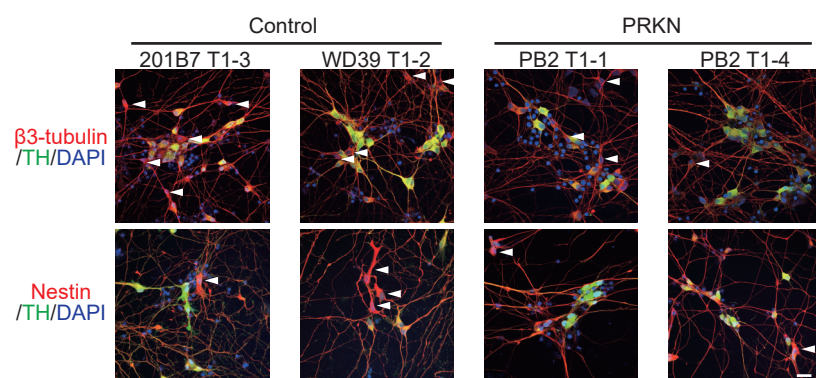

Supplement: Supplementary file 2 — Additional file 2: Figure S1. Characterization of non-dopaminergic neurons differentiated from TH-GFP iPSC lines. Immunofluorescence staining for TH, β3-tubulin, and Nestin identified other neural subtypes (β3-tubulin + TH-) and neural progenitor cells (Nestin + TH-) among the non-dopaminergic cells derived from TH-GFP iPSC lines. Arrowheads indicate β3-tubulin + TH- or Nestin + TH- cells. “PRKN” represents PRKN-mutated patient. Scale bar, 20 µm. [file 13041_2021_771_MOESM2_ESM.pdf]

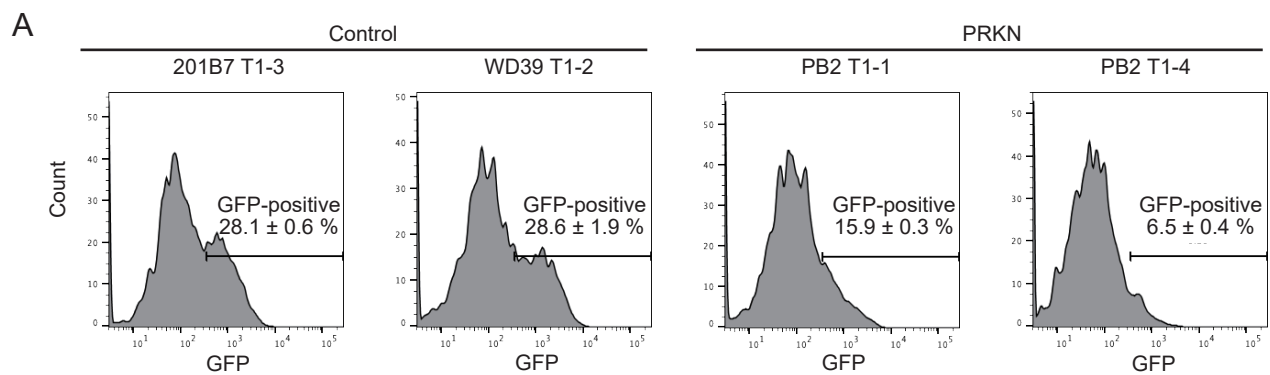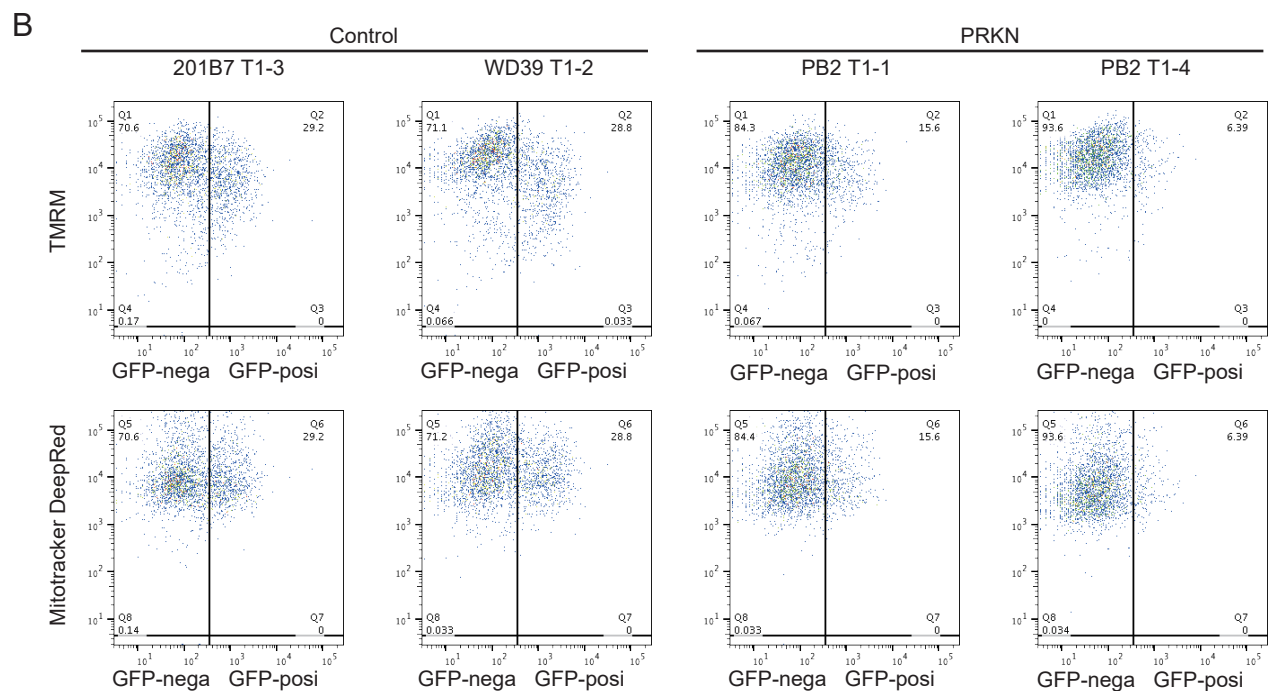

Supplement: Supplementary file 3 — Additional file 3: Figure S2. Flow cytometric analysis of mitochondrial membrane potential in dopaminergic neurons derived from TH-GFP iPSCs. (a) Histograms showed GFP-negative and GFP-positive populations in the TH-GFP iPSC-derived differentiated cells. Data were obtained from three independent experiments. “PRKN” represents PRKN-mutated patient. Values are shown as the mean ± SEM. (b) TMRM-GFP cytograms (top) represented the intensity of TMRM signal in GFP-negative and -positive populations. Mitotracker DeepRed-GFP cytograms (bottom) represented the intensity of Mitotracker DeepRed signal in GFP-positive and -positive populations. “PRKN” represents PRKN-mutated patient. (c) Quantitative analysis of the MFI of TMRM (left), Mitotracker DeepRed (center), and TMRM normalized with Mitotracker DeepRed (right) in GFP-positive cells relative to GFP-negative cells in the control and PRKN-mutated lines. Data were obtained from three independent experiments. “PRKN” represents PRKN-mutated patient. Values are shown as the mean ± SEM. Statistical significance was evaluated using the unpaired two-tailed t-test. *P < 0.05. There was no significant difference in the MFI of Mitotracker between control GFP-positive and -negative cells (control center; P = 0.3502). There were no significant differences in the MFI of TMRM and TMRM/Mitotracker between PRKN-mutated GFP-positive and -negative cells (PRKN left; P = 0.5567, PRKN right; P = 0.1382). [file 13041_2021_771_MOESM3_ESM.pdf]

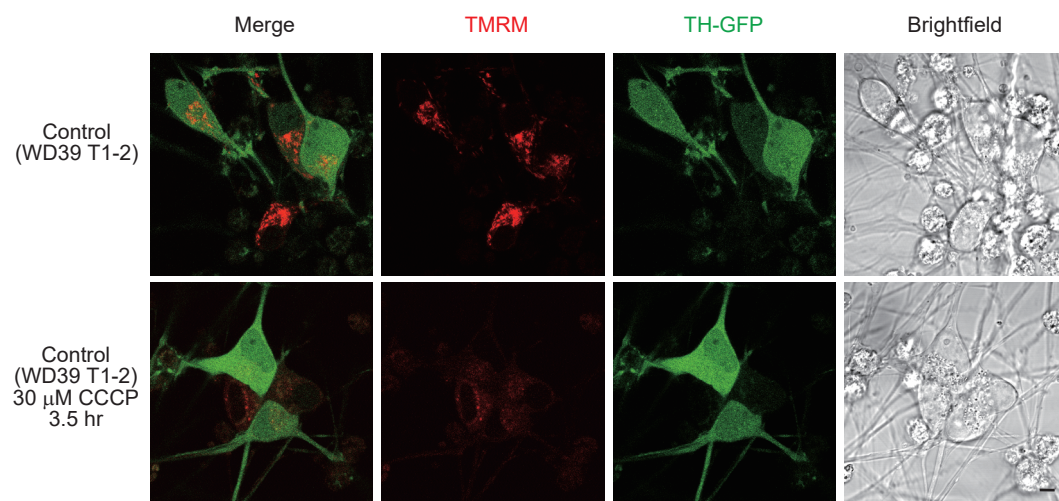

Supplement: Supplementary file 4 — Additional file 4: Figure S3. Mitochondrial membrane potential in iPSC-derived cells under CCCP treatment. Mitochondrial membrane potential in dopaminergic and non-dopaminergic neurons derived from control TH-GFP iPSCs under normal conditions and CCCP treatment. CCCP treatment for 3.5 h rapidly attenuated the fluorescence of TMRM. Scale bar, 5 µm. [file 13041_2021_771_MOESM4_ESM.pdf]
